# Intraday and Interday Reliability of Maximal and Explosive Handgrip Force–Time Metrics Using the Kinvent K-Grip Handheld Dynamometer

**DOI:** 10.3390/muscles5020024

**Published:** 2026-03-25

**Authors:** Ivan Curovic, Milan Markovic, Lazar Toskic, Jill Alexander, Damian J. Harper

**Affiliations:** 1 Institute of Coaching and Performance, School of Health, Social Work and Sport, University of Lancashire, Preston PR1 2HE, UK; 2 Faculty of Sport and Physical Education, University of Priština in Kosovska Mitrovica, 38218 Leposavić, Serbia; 3 Faculty of Sport, University ‘’Union-Nikola Tesla’’, 11000 Belgrade, Serbia

**Keywords:** rate of force development, impulse, neuromuscular function, explosive force, strength assessment

## Abstract

(1) Background: Handgrip strength (HGS) is a widely used indicator of neuromuscular function, with predictive values for health and performance outcomes. The aim of this study was to evaluate the intraday and interday reliability of maximal and explosive handgrip force–time metrics using the Kinvent K-Grip handheld dynamometer. (2) Methods: Thirty-four participants performed three maximal voluntary isometric contractions per hand across two testing days. Force–time data were analysed for peak force (PF), mean force (MF), peak rate of force development (RFD), time-specific RFD, impulse, and forces at fixed time points. Reliability was assessed using intraclass correlation coefficients (ICCs), standard error of measurement (SEM), minimal detectable change (MDC), and coefficient of variation (CV%). (3) Results: The device demonstrated excellent relative and absolute reliability for PF and MF across both days (ICC > 0.97; CV < 6%; MDC ≈ 5 kg). Later-phase explosive metrics (F250 and Imp200) showed good-to-excellent relative reliability (ICC = 0.88-0.99; CV = 4–14%), although with variable absolute reliability (MDC F250 ≈ 4–8 kg, MDC Imp200 ≈ 1 kg·s). For early-phase metrics, relative reliability was only moderate to good (ICC = 0.67–0.88) and characterised by a high degree of variability (CV = 15–22%). (4) Conclusions: The K-Grip handheld dynamometer is a reliable tool for cross-sectional assessments and for tracking larger maximal strength and later-phase force improvements at fixed time points. Early-phase explosive metrics are less suitable for monitoring intervention effects due to high measurement error and fatigue sensitivity.

## 1. Introduction

The process of measuring and evaluating the performance and health status of an individual is an important part of sports training and clinical practice. Handgrip dynamometers are some of the most common, easily accessible and portable tools used to assess muscular strength [1] and neuromuscular function [2] in clinical and performance-based contexts [3,4,5]. While low handgrip strength (HGS) has been consistently associated with increased risks of all-cause, cancer-related, and cardiovascular mortality across sexes and age groups [6], greater HGS has emerged as a strong, independent predictor of longevity and health [6,7]. Indeed, weak HGS is linked to increased hospitalisation, poor nutritional status, reduced quality of life and overall mortality, as well as a higher incidence of various diseases including cancer, diabetes, chronic kidney and liver disease [5]. On the other hand, a higher level of HGS appears to be a protective mechanism that supports cognitive function and functional independence among older adults [4,8]. Likewise, HGS has been extensively used in sports diagnostics to reflect the upper body strength capacity [1,9,10] and neuromuscular potential of an athlete [2,11,12].

The emergence and significance of measuring rapid force-generating characteristics such as the rate of force development (RFD) with modern handgrip dynamometers mark a substantial evolution in practical neuromuscular assessment [2,10]. While maximal isometric strength indicates the ceiling of force production, RFD reflects the ability of the nervous system to recruit motor units rapidly and synchronise their firing from the very onset of contraction [13], commonly referred to as ‘explosive’ strength [14]. This strength quality is a primary determinant of success in sports requiring rapid limb movement with high-intensity actions [15]. HGS, therefore, does not only serve as a simple measure of forearm strength but also acts as a practical and globally accessible proxy for overall neuromuscular and explosive potential. This is supported by studies demonstrating not only the expected correlation between general upper body strength and handgrip maximal force [1,9,10] but also significant associations between greater handgrip force-generating metrics such as RFD and lower-body performance measured by vertical jump and agility tests [2,11,16], suggesting that handgrip explosive metrics may reflect broader neuromuscular function [2,11,16,17].

Measuring explosive handgrip metrics like RFD could offer a highly practical, time-efficient and non-fatiguing method to estimate critical athletic strength qualities that are directly related to sports success, providing a valuable tool for talent identification [3], monitoring training interventions [9], and profiling athletes across a wide range of sports [18]. Critically, the physiological determinants of force production differ across the time course of contraction: early-phase RFD (0–50, 0–100 ms) is primarily governed by neural factors such as motor unit recruitment and discharge rate, whereas later-phase force development (150–200 ms) increasingly reflects maximal strength capacity and muscle cross-sectional area [13,15]. Furthermore, contractile impulse, defined as the force–time integral during the initial phase of contraction [13], provides insight into the total explosive force accumulated and is closely linked to neural drive [15]. Understanding the reliability of metrics across these distinct phases is therefore essential for determining whether handgrip dynamometry can validly capture both neural and maximal strength qualities.

The utility of handgrip dynamometry in clinical and athletic domains hinges on the reliability of the measurements it produces [19]. Across the literature, the test–retest reliability of traditional handgrip strength metrics, such as peak force, has been shown to be excellent, with intraclass correlation coefficients (ICCs) frequently exceeding 0.90 [18,20,21]. Less attention, however, has been given to the reliability of time-dependent force metrics, such as RFD and contractile impulse [18], despite their growing relevance for assessing neuromuscular performance. A limited number of investigations indicate that RFD exhibits excellent test–retest reliability, with ICCs typically reported in the high range (e.g., 0.89–0.98) [2,22], supporting the use of handheld dynamometers as a reliable measure of explosive force characteristics. Nevertheless, it is important to note that the body of evidence supporting these high reliability coefficients for time-dependent metrics is not yet extensive or robust [18]. While the existing findings are promising, they are often derived from studies with relatively small sample sizes (<25) and homogenous populations (i.e., females) [2,22], utilising different handgrip devices.

Since the number of manufacturers of sports diagnostics equipment is increasing, there is a need for continuous investigation of the reliability and usability of these tools. Accordingly, several studies have investigated the validity and reliability of the novel Kinvent K-Grip, a Bluetooth-enabled handgrip dynamometer that provides real-time digital force measurements. For instance, Nikodelis et al. [23] showed that the K-Grip produced highly reliable maximal force measurements in both clinical and healthy populations, with strong agreement when compared to the Jamar device [23]. Similarly, van Iperen et al. [24] reported excellent intraday and interday reliability of the K-Grip in critically ill patients, with ICCs ranging from 0.94 to 0.99, and perfect agreement with calibrated weights (r = 1.0), supporting its use even for very weak, critically ill patients recovering from intensive care [24]. In another evaluation, Almeida et al. [21] confirmed good-to-excellent reliability of the Kinvent device across multiple upper and lower limb muscle groups, aligning with COSMIN guidelines. Despite these promising results, all these studies focused exclusively on maximal force output, with no data available on the reliability of explosive force–time metrics such as RFD, contractile impulse, or forces at fixed time points. This represents a critical gap in the literature, especially given the increasing use of handgrip testing in athletic and performance contexts where rapid force generation is highly relevant [18]. The present study therefore aims to examine the interday and intraday reliability of the Kinvent K-Grip handheld dynamometer across a broad range of force metrics, offering a more comprehensive assessment of its utility.

## 2. Methods and Materials

### 2.1. Participants

Thirty-four recreationally active adults (mean ± SD; age = 23 ± 4.14 years; 23 males, 11 females) voluntarily participated in this study. This sample size with three trials per session aligns with the recommendations for efficient precision in reliability studies when expected ICC values are higher than expected [25]. All participants were free from injuries or neuromuscular disorders that could affect grip performance. Recreationally active was defined as engaging in moderate-to-vigorous physical activity at least three times per week. Participants provided written informed consent prior to participation, and all procedures were approved by the University of Lancashire’s ethics committee (HEALTH 0330).

### 2.2. Instrumentation

Handgrip force data were collected using the K-Grip dynamometer (Kinvent Biomechanics, Montpellier, France; K-Grip V3, model KMN/FT-JT, serial number PG122061), a wireless, portable handgrip device equipped with a high-frequency (1000 Hz) force sensor and Bluetooth data capture. The Kinvent app (version 2.23.0) was used to visualise and export force–time curves for each trial.

### 2.3. Procedure

Testing was conducted on two separate days, spaced 48 h apart, at approximately the same time of day to minimise diurnal variation [21]. Participants refrained from strenuous physical activity for 24 h prior to each session. For each testing day, subjects performed three maximal voluntary contractions with each hand, totalling six trials per day. Each contraction lasted for 3 s, with a standardised one-minute rest period between trials to minimise fatigue [18]. All three trials were completed with the right hand first, followed by all three trials with the left hand. This sequence was implemented to control for potential cross-transfer effects, ensuring that any fatigue or neuromuscular priming from the preceding contractions would consistently affect the tested hand, thereby standardising the order effect across all participants. Participants were seated upright in a chair with their elbow flexed at 90°, forearm in a neutral position, and wrist slightly extended [18]. Warm-up trials involved 3 submaximal contractions at approximately 50%, 75%, and 90% of perceived maximum force to adequately prepare the neuromuscular system for testing without inducing fatigue [26]. Verbal encouragement was provided during each trial to promote maximal effort. Importantly, all participants were instructed to “squeeze the handle as hard and as fast as possible to produce maximal force with maximal explosiveness from the very start of the contraction.” This emphasis on both peak effort and rapid force generation ensured that measurements captured not only maximal voluntary force capacity but also the participants’ ability to develop force quickly [14].

### 2.4. Data Extraction and Force Metrics

From the force–time data collected during each contraction, a comprehensive set of force–time metrics were extracted using Kinvent Physio App (version 2.23.0) and further processed in Microsoft Excel (Table 1). Force–time signals were processed internally by the Kinvent software prior to export, and no additional filtering or smoothing was applied during external analysis. Contraction onset was determined automatically by the Kinvent proprietary software using a threshold-based algorithm.

### 2.5. Statistical Analysis

Intraday reliability was evaluated using data from all three trials on a single testing day. Relative reliability was determined using two-way random-effects and the absolute-agreement intraclass correlation coefficient (ICC_2_ₖ) [27] as trials were considered randomly selected from all possible trials and absolute value consistency was required. The corresponding standard error of measurement (SEM) was derived from the ICC output using the formula: $\mathrm{SEM}=\mathrm{Pooled Standard Deviation}\times \sqrt{\left(1-\mathrm{ICC}\right)}$ [28], which leverages variance components across the three trials within a single session.

Interday reliability was assessed by comparing the average scores from day 1 and day 2. A two-way mixed-effects, consistency ICC (ICC_3_ₖ) [27] was used, as the primary interest was whether participants maintained their relative ranking across days despite potential systematic shifts (e.g., familiarisation, circadian variation). Although absolute agreement models are often recommended when strict reproducibility of absolute values is the primary objective, a consistency model was selected here because the primary interest was whether individuals maintained their relative ranking across testing days. The SEM was then calculated using the standard deviation of the difference scores between day 1 and day 2 as $\mathrm{SEM}=\mathrm{SD of Difference Scores}/\sqrt{2}$ [29] to directly capture day-to-day biological and technical variation using the standard deviation of difference scores between the two testing days. This interday SEM was used to calculate the minimal detectable change (MDC) at a 95% confidence level: $\mathrm{MDC}=1.96\times \sqrt{2}\times \mathrm{SEM}$ [28].

ICC values were interpreted as follows: <0.50 poor, 0.50–0.75 moderate, 0.75–0.90 good, and >0.90 excellent [27]. CV% values were classified as: <5% excellent, 5–10% good, 10–15% moderate, and >15% poor. These thresholds are consistent with established conventions in sports medicine and reliability research [29], where CV <10% is generally considered acceptable for performance tests. To evaluate systematic changes, paired-sample t-tests were used to compare mean scores between the days. Repeated-measures ANOVA was employed to test for significant trial effects within each day. The threshold for statistical significance was set at *p* < 0.05. All statistical analyses were performed using SPSS (version 28.0; IBM Corp., Armonk, NY, USA).

## 3. Results

### 3.1. Intraday Reliability

Intraday reliability for the first tested (right) and second tested (left) hand is illustrated in Table 2 and Table 3, respectively. Peak and mean force demonstrated excellent reliability for both hands, with ICCs > 0.98, CV% ranging from 2.8% to 3.3%, and low SEM values (1.03–1.17 kg), confirming the high stability of these measurements within a session. Repeated-measures ANOVA revealed no significant trial effects for any variable (*p* > 0.05; see Supplementary Table S1).

The reliability of explosive force metrics was more variable. Peak RFD showed good-to-excellent reliability (right-hand: ICC = 0.82, CV = 10.9%; left-hand: ICC = 0.92, CV = 7.3%). However, the reliability of time-specific RFD metrics decreased as the time window increased and was generally lower for the right hand (ICC = 0.61–0.75; CV = 15–17%) compared to the left (ICC = 0.74–0.90; CV = 7–13%). A similar pattern was observed for contractile impulse (ICC = 0.75–0.91; CV = 9–13%) and early-phase force values (ICC = 0.83–0.92; CV = 8–12%), which showed good-to-excellent reliability, with the second-tested hand (left) again demonstrating higher consistency. Force at 250 ms (F_250_) was a notable exception, exhibiting excellent reliability (ICCs > 0.95) and low variability (CV% < 5.6%) for both hands.

### 3.2. Interday Reliability

Interday reliability for the right and left hands is illustrated in Table 4 and Table 5, respectively. Mirroring the intraday findings, maximal strength variables (i.e., peak and mean force) exhibited high interday scores. Relative reliability was excellent, with ICCs > 0.96 for all measures. In regard to absolute reliability and measurement error, the CV% was good (< 5.8%) and the MDC for peak force was approximately 5.5 kg for both hands, representing 15.2% and 15.7% of the mean peak force for the right and left hands, respectively.

For explosive metrics, interday reliability was generally lower than intraday reliability. The first tested (right) hand demonstrated good relative reliability for most variables (ICCs = 0.80–0.90), though corresponding CV% values were higher, often in the poor-to-moderate range (e.g., 13–18%). Values for early-phase RFD were lower, reaching moderate levels for ICC (e.g., RFD0-150, ICC = 0.80) and poor levels for CV% (e.g., RFD0-150, CV = 15.9%). The second tested (left) hand showed greater variability, with moderate relative reliability for most RFD metrics (ICCs = 0.63–0.74 for Peak RFD, RFD0-100, RFD0-150, and RFD0-200) and poor associated absolute reliability (CV% = 17–22%), but good reliability for RFD0-50 (ICC = 0.76). In contrast, both hands maintained excellent relative reliability for time-specific forces (F_50_, F_100_, F_250_) and later-phase impulse (Imp_0–200_) (ICCs > 0.88, mostly > 0.93), which was matched by good-to-excellent absolute reliability (CV% = 4–11%). Paired-sample t-tests revealed no significant systematic differences between days for any variable for either hand (*p* > 0.05; see Supplementary Table S1), indicating no learning or fatigue effect across the 48 h period.

## 4. Discussion

The current study aimed to provide a comprehensive, multi-day evaluation of the Kinvent K-Grip dynamometer’s reliability for assessing maximal and explosive handgrip forces. The primary findings reveal a clear hierarchy of measurement robustness that is critically dependent on the specific metric and its neuromuscular demands. Maximal strength (peak and mean force) demonstrated excellent reliability (ICC > 0.97), confirming the device’s high precision for assessing this capacity. In contrast, the reliability of explosive metrics was strongly phase-dependent. While later-phase measures, such as the force at 250 ms and impulse at 0–200 ms, showed good-to-excellent relative reliability (ICC = 0.88–0.99), early-phase RFD and impulse metrics exhibited only good-to-moderate reliability (ICC = 0.67–0.88) and high absolute variability (CV = 15–22%). Analysis of measurement error and MDC revealed that while maximal strength metrics retain practical utility for tracking larger individual changes, the explosive metrics may lack the necessary precision to detect meaningful changes at the individual level in intervention studies.

The current findings regarding maximal force correspond strongly with previous studies [21,23]. For example, Nikodelis et al. [23] reported high test–retest reliability and validity against the gold-standard Jamar dynamometer, albeit with noted differences in absolute values between the two devices. This aligns with our high ICCs for peak force and mean force, reinforcing the device’s established role in assessing maximal strength in healthy [23] and clinical populations [24]. Furthermore, the excellent interday reliability reported by Almeida et al. [21] across multiple muscle groups is consistent with the strong interday reliability we observed for most variables. However, the existing literature on the K-Grip has, until now, been exclusively limited to the maximal force output. The current study directly addresses this gap and reveals that reliability with handgrip testing is phase-dependent. The lower ICCs we observed for early-phase metrics are consistent with their greater sensitivity to neural drive [13,15], a finding previously noted by Jenkins et al. [30] with an older cohort.

The high variability in reliability scores and CVs for early explosive metrics observed in this study is aligned with previous research, where reported reliability for RFD has ranged from poor to excellent depending on the specific metric, population, and protocol used [2,22,30]. In our investigation, reliability decreased for very early-phase RFD metrics (e.g., 0–50, 0–100 ms), a finding that likely reflects their increased neural complexity [15]. Furthermore, we identified a significant order effect: the second-tested (left) hand demonstrated superior intraday reliability for explosive metrics compared to the first-tested (right) hand. However, this greater within-session consistency should not be interpreted as a true practice benefit in terms of enhanced performance. Rather, as shown in Table 3, the left hand’s initial trial values were substantially lower than the right hand’s fresh values (e.g., peak RFD: 203.74 vs. 268.24 kg/s), suggesting that supraspinal fatigue from the preceding maximal efforts [31] attenuated explosive capacity from the outset. This fatigue-induced ceiling effect resulted in a less-pronounced decline across the left hand’s three trials, artificially inflating within-session consistency while actually reflecting a depressed neuromuscular state. Indeed, repeated maximal efforts are known to depress early-phase force generation [13], which could affect subsequently activated muscles via supraspinal fatigue [31]. Previous research has shown that when grip flexors are fatigued to 60–40% of their maximal forces, the RFD decreases by up to 50%, and the time to reach 50–70% of maximal force is significantly prolonged [32]. Importantly, this within-session stability did not persist across days (i.e., the left hand showed greater interday variability), confirming that the apparent ‘practice effect’ was transient and fatigue-mediated rather than a durable learning effect. These findings emphasise that the reliability of explosive metrics is highly vulnerable to neuromuscular fatigue, and that apparent within-session consistency may sometimes mask underlying fatigue rather than reflect true measurement stability. This suggests that when assessing explosive handgrip metrics, researchers should consider both the order of limb testing and the adequacy of rest periods, with our data indicating that one minute may be insufficient [18].

While maximal grip strength is a potent health biomarker [5,6,7], explosive metrics like RFD and contractile impulse provide a unique window into the quality of force production and the integrity of the neuromuscular system [13,15]. These metrics reflect the ability to rapidly recruit high-threshold motor units, which is a key determinant of functional capacity [2,26,33,34]. The significance of measuring them goes beyond the assessment of an upper-extremity force production, but may represent the whole-body explosive capacity, including the lower extremities [2,33,34,35], implying a shared central motor command [17]. For example, Maurya et al. [2] found handgrip RFD to be a reliable measure that correlated significantly with lower-body power outputs, supporting its use as a proxy for athletic capacity in settings where more complex testing is impractical. Our data confirms that a modern, accessible device like the K-Grip can reliably capture these qualities, although translating this potential into real-world application requires consideration of the absolute reliability findings. While ICC values indicate that the K-Force Grip can consistently rank individuals for explosive strength, the absolute measurement error for these metrics was substantial. CV% values often exceeded 15–20%, limiting the device’s sensitivity for detecting small but meaningful changes within individuals. This suggests that although the device may distinguish between novices and elite performers, it may not be precise enough to track short-term training adaptations in early-phase metrics. In contrast, maximal strength and later-phase force outputs demonstrated lower absolute error, thereby supporting their use in both cross-sectional assessment and longitudinal monitoring. Clinically, an MDC of 5.5 kg for peak force should be interpreted relative to established minimal clinically important differences, which for handgrip strength range from 5 to 6.5 kg [36]. Therefore, the MDC observed in the present study falls within a range where meaningful changes in maximal strength could be detected.

This study is not without limitations. First, the sample consisted exclusively of recreationally active young adults, which limits the generalisability of the findings to other populations such as older individuals, clinical patients, or elite athletes, whose neuromuscular function and fatigability may differ substantially. Second, the protocol mandated that the right hand was always tested first, rather than employing a randomised or counterbalanced order. While this may have introduced a systematic order effect, it was an intentional methodological choice to rigorously test for the presence of fatigue induced by the standardised one-minute rest period. Consequently, the observed asymmetry in fatigue and reliability between the first-tested and second-tested limb is a direct outcome of this design. Third, the contraction onset was determined automatically using the proprietary Kinvent algorithm. Although the same automated procedure was consistently applied across all trials and sessions, the proprietary nature of the algorithm prevents direct verification of the detection criteria. Given that early-phase RFD is sensitive to onset determination, minor variability in early explosive metrics may partially reflect automated onset estimation. However, because the same proprietary filtering was applied consistently across all trials and sessions, any potential influence on reliability estimates is uniform and does not compromise within-study comparisons. Finally, because the aim of the present study was to evaluate device reliability across a heterogeneous recreational sample, pooling sexes was considered appropriate for this reliability objective. However, future studies with larger and sex-balanced samples should investigate whether reliability patterns differ between males and females.

## 5. Conclusions

This study provides a comprehensive reliability analysis of handgrip force metrics, revealing a clear hierarchy of measurement robustness. Our findings establish that the device is highly suited for assessing maximal strength (peak and mean force) and later-phase explosive forces (Imp_200_, F_250_). However, its utility for early-phase explosive metrics is constrained by significant fatigue sensitivity and high measurement error. Consequently, we propose three primary recommendations for practice: (1) To optimise testing protocols, future studies should employ counterbalanced limb order or extended rest periods (>1 min) to mitigate the fatigue-induced variability that undermines the reliability of explosive metrics. (2) For ranking individuals in cross-sectional studies, the device is effective for maximal strength qualities and later-phase force indices, as well as for intra-session explosive metrics. (3) For monitoring individual progress over time, practitioners should prioritise peak force, mean force, force at 250 ms, and impulse in 0–200 ms, as early-phase explosive metrics may lack the necessary precision to detect meaningful change.

## Figures and Tables

**Table 1:** Handgrip force–time metrics obtained from the Kinvent K-Grip handheld dynamometer.

Metric	Unit	Definition
Peak force	kg	Maximum force exerted during test
Mean force	kg	Average force maintained during the contraction phase
Time-specific RFD	kg/s	Average rate of force increase from onset to each time point
Time-specific impulse	kg·s	Total force applied over the specified time interval (area under the curve) *
Time-specific forces	kg	Force value recorded at each corresponding time after onset

RFD = rate of force development. * Note: In this study, ‘impulse’ refers to the force–time integral (area under the force–time curve) during the specified epoch, a term commonly used in isometric force–time analysis to denote total accumulated force [13].

**Table 2:** Descriptive statistics, intraday reliability, and sensitivity of K-Grip dynamometer force–time variables for the right hand.

	Mean per Trial			Standard Error		Intraclass Coefficient			Coefficient of Variation	
Variables	Trial	Mean	SD	SEM	95% CI	ICC	95% CI	Rating	CV%	95% CI
Peak force (kg)	1	37.88	8.36	1.03	0.83–1.38	0.984	0.971–0.991	Excellent	2.78	2.23–3.73
	2	37.15	8.08							
	3	35.95	8.18							
Mean force (kg)	1	33.09	7.32	1.10	0.88–1.48	0.979	0.964–0.989	Excellent	3.27	2.63–4.39
	2	34.47	7.9							
	3	33.27	8.29							
Peak RFD (kg/s)	1	268.24	86.04	23.84	19.14–32.02	0.824	0.688–0.906	Good	10.89	8.74–14.63
	2	204.97	42.35							
	3	183.4	62.3							
RFD_0–50 (kg/s)	1	162.78	92.14	28.32	22.74–38.03	0.744	0.548–0.864	Moderate	16.77	13.47–22.52
	2	177.7	46.97							
	3	166.17	59.27							
RFD_0–100 (kg/s)	1	191.62	84.78	25.52	20.49–34.27	0.696	0.464–0.838	Moderate	15.35	12.33–20.62
	2	160.74	33.43							
	3	146.51	45.14							
RFD_0–150 (kg/s)	1	166.8	63.88	20.41	16.39–27.41	0.639	0.362–0.808	Moderate	15.10	12.13–20.28
	2	125.39	25.48							
	3	113.29	35.12							
RFD_0–200 (kg/s)	1	138.11	49.75	16.67	13.39–22.39	0.611	0.312–0.793	Moderate	14.99	12.04–20.13
	2	101.2	21.49							
	3	94.24	29.54							
Imp_0–50 (kg·s)	1	0.28	0.11	0.09	0.07–0.12	0.754	0.565–0.869	Good	13.43	10.78–18.04
	2	0.85	0.24							
	3	0.88	0.29							
Imp_0–150 (kg·s)	1	1.12	0.48	0.20	0.16–0.27	0.872	0.773–0.932	Good	10.75	8.63–14.44
	2	2.23	0.64							
	3	2.24	0.73							
Imp_0–200 (kg·s)	1	2.31	0.92	0.31	0.25–0.42	0.867	0.765–0.929	Good	9.54	7.66–12.81
	2	3.74	0.92							
	3	3.69	1.07							
Force_50 (kg)	1	10.78	4.98	2.16	1.73–2.9	0.834	0.706–0.911	Good	11.51	9.24–15.46
	2	22.7	6.14							
	3	22.8	7.02							
Force_100 (kg)	1	21.64	9.03	2.70	2.17–3.63	0.859	0.751–0.925	Good	10.06	8.08–13.51
	2	29.91	6.97							
	3	28.96	8.32							
Force_250 (kg)	1	32.86	9.21	1.85	1.49–2.48	0.953	0.916–0.975	Excellent	5.57	4.47–7.48
	2	33.96	8.27							
	3	32.87	9.23							

SD = standard deviation; SEM = standard error of measurement; CI = confidence interval; ICC = intraclass correlation coefficient; CV = coefficient of variation; RFD = rate of force development; Imp = impulse.

**Table 3:** Descriptive statistics, intraday reliability, and sensitivity of K-Grip handgrip force–time variables for the left hand.

	Mean per Trial			Standard Error		Intraclass Coefficient			Coefficient of Variation	
Variables	Trial	Mean	SD	SEM	95% CI	ICC	95% CI	Rating	CV%	95% CI
Peak force (kg)	1	37.06	8.22	1.17	0.94–1.57	0.980	0.964–0.989	Excellent	3.23	2.59–4.34
	2	36.11	8.46							
	3	35.5	8.3							
Mean force (kg)	1	34.25	7.95	1.12	0.9–1.5	0.980	0.964–0.989	Excellent	3.33	2.67–4.47
	2	33.61	8.36							
	3	32.92	7.96							
Peak RFD (kg/s)	1	203.74	57.99	14.46	11.61–19.42	0.921	0.860–0.958	Excellent	7.34	5.89–9.86
	2	193.21	58.0							
	3	193.68	49.5							
RFD_0–50 (kg/s)	1	172.01	60.64	20.80	16.7–27.93	0.857	0.747–0.924	Good	12.64	10.15–16.98
	2	164.77	63.98							
	3	157.06	62.39							
RFD_0–100 (kg/s)	1	161.04	45.39	15.97	12.82–21.45	0.841	0.72–0.915	Good	10.48	8.42–14.07
	2	151.95	46.25							
	3	144.04	46.36							
RFD_0–150 (kg/s)	1	125.91	33.04	10.49	8.42–14.09	0.861	0.754–0.926	Good	8.67	6.96–11.64
	2	121.41	32.07							
	3	115.52	30.15							
RFD_0–200 (kg/s)	1	102.69	24.99	7.03	5.65–9.44	0.897	0.817–0.945	Good	7.10	5.7–9.54
	2	99.29	24.58							
	3	95.25	22.38							
Imp_0–50 (kg·s)	1	0.83	0.31	0.10	0.08–0.13	0.866	0.763–0.929	Good	12.05	9.68–16.18
	2	0.85	0.32							
	3	0.8	0.24							
Imp_0–150 (kg·s)	1	2.17	0.74	0.20	0.16–0.27	0.905	0.833–0.95	Excellent	9.30	7.47–12.49
	2	2.2	0.76							
	3	2.07	0.65							
Imp_0–200 (kg·s)	1	3.67	1.15	0.30	0.24–0.4	0.907	0.836–0.95	Excellent	8.31	6.67–11.16
	2	3.69	1.12							
	3	3.49	0.98							
Force_50 (kg)	1	22.31	7.12	2.04	1.64–2.74	0.905	0.832–0.949	Excellent	9.32	7.48–12.52
	2	22.39	7.69							
	3	20.94	6.77							
Force_100 (kg)	1	29.67	8.21	2.18	1.75–2.93	0.919	0.856–0.957	Excellent	7.57	6.08–10.17
	2	29.18	8.48							
	3	27.53	8.0							
Force_250 (kg)	1	35.07	7.67	1.31	1.05–1.76	0.971	0.948–0.984	Excellent	3.82	3.07–5.13
	2	34.12	8.1							
	3	33.6	7.75							

SD = standard deviation; SEM = standard error of measurement; CI = confidence interval; ICC = intraclass correlation coefficient; CV = coefficient of variation; RFD = rate of force development; Imp = impulse.

**Table 4:** Descriptive statistics, reliability metrics and comparative analysis of variables between the days of examination for the right hand.

	Mean per Trial		Absolute Reliability			Relative Reliability		Intraclass Coefficient		
Variables	Day	Mean ± SD	SEM	95% CI	MDC	CV%	95% CI	ICC	95% CI	Rating
Peak force (kg)	I	36.99 ± 8.08	1.98	1.59–2.66	5.49	5.39	4.33–7.24	0.969	0.938–0.985	Excellent
	II	36.59 ± 8.09								
Mean force (kg)	I	33.61 ± 7.69	1.86	1.5–2.5	5.17	5.58	4.48–7.49	0.969	0.938–0.985	Excellent
	II	33.26 ± 7.56								
Peak RFD (kg/s)	I	218.87 ± 56.77	27.10	21.76–36.39	75.06	12.65	10.16–16.99	0.893	0.786–0.947	Good
	II	209.55 ± 66.3								
RFD_0–50 (kg/s)	I	168.89 ± 55.97	32.74	26.29–43.96	90.68	20.18	16.2–27.1	0.832	0.664–0.916	Good
	II	155.57 ± 65.81								
RFD_0–100 (kg/s)	I	166.29 ± 46.31	29.45	23.65–39.55	81.58	18.39	14.77–24.69	0.799	0.598–0.900	Good
	II	154.04 ± 55.19								
RFD_0–150 (kg/s)	I	135.16 ± 33.97	21.05	16.9–28.27	58.31	15.93	12.79–21.39	0.795	0.589–0.897	Good
	II	129.19 ± 38.03								
RFD_0–200 (kg/s)	I	111.18 ± 26.72	14.54	11.68–19.53	40.29	13.27	10.66–17.83	0.825	0.65–0.913	Good
	II	107.96 ± 26.62								
Imp_0–50 (kg·s)	I	0.67 ± 0.18	0.10	0.08–0.13	0.27	15.21	12.21–20.43	0.855	0.709–0.927	Good
	II	0.64 ± 0.21								
Imp_0–150 (kg·s)	I	1.86 ± 0.56	0.32	0.26–0.43	0.89	17.85	14.33–23.97	0.804	0.608–0.902	Good
	II	1.74 ± 0.56								
Imp_0–200 (kg·s)	I	3.25 ± 0.86	0.43	0.34–0.57	1.19	13.46	10.81–18.07	0.875	0.751–0.938	Good
	II	3.11 ± 0.95								
Force_50 (kg)	I	18.76 ± 5.29	2.69	2.16–3.61	7.45	14.69	11.8–19.73	0.871	0.742–0.936	Good
	II	17.87 ± 5.95								
Force_100 (kg)	I	26.84 ± 7.2	3.61	2.9–4.85	10.00	13.76	11.05–18.48	0.870	0.740–0.935	Good
	II	25.65 ± 7.86								
Force_250 (kg)	I	33.23 ± 8.52	2.81	2.26–3.78	7.79	8.50	6.82–11.41	0.936	0.873–0.968	Excellent
	II	32.92 ± 7.71								

SD = standard deviation; SEM = standard error of measurement; CI = confidence interval; ICC = intraclass correlation coefficient; CV = coefficient of variation; RFD = rate of force development; Imp = impulse.

**Table 5:** Descriptive statistics, reliability metrics and comparative analysis of variables between the days of examination for the left hand.

	Mean per Trial		Absolute Reliability			Relative Reliability		Intraclass Coefficient		
Variables	Day	Mean ± SD	SEM	95% CI	MDC	CV%	95% CI	ICC	95% CI	Rating
Peak force (kg)	I	36.23 ± 8.16	2.05	1.65–2.75	5.68	5.58	4.48–7.5	0.969	0.938–0.984	Excellent
	II	37.21 ± 8.53								
Mean force (kg)	I	33.59 ± 7.93	1.97	1.59–2.65	5.47	5.79	4.65–7.78	0.970	0.940–0.985	Excellent
	II	34.59 ± 8.38								
Peak RFD (kg/s)	I	196.87 ± 51.39	38.70	31.07–51.97	107.19	20.47	16.43–27.49	0.698	0.396–0.849	Moderate
	II	181.27 ± 61.8								
RFD_0–50 (kg/s)	I	164.61 ± 54.98	34.88	28.01–46.84	96.61	22.11	17.75–29.69	0.758	0.515–0.879	Good
	II	150.94 ± 56.68								
RFD_0–100 (kg/s)	I	152.35 ± 40.08	31.64	25.41–42.5	87.66	21.59	17.34–29	0.671	0.341–0.836	Moderate
	II	140.76 ± 49.37								
RFD_0–150 (kg/s)	I	120.95 ± 28.1	24.78	19.9–33.29	68.65	21.26	17.07–28.55	0.632	0.263–0.816	Moderate
	II	112.24 ± 38.64								
RFD_0–200 (kg/s)	I	99.08 ± 21.86	16.23	13.03–21.79	44.95	16.87	13.55–22.66	0.738	0.474–0.869	Moderate
	II	93.25 ± 28.08								
Imp_0–50 (kg·s)	I	0.83 ± 0.26	0.13	0.11–0.18	0.37	15.60	12.52–20.95	0.881	0.761–0.94	Good
	II	0.88 ± 0.32								
Imp_0–150 (kg·s)	I	2.15 ± 0.66	0.31	0.25–0.41	0.85	14.15	11.36–19	0.884	0.768–0.942	Good
	II	2.2 ± 0.69								
Imp_0–200 (kg·s)	I	3.61 ± 0.99	0.32	0.26–0.43	0.89	8.72	7–11.71	0.950	0.899–0.975	Excellent
	II	3.75 ± 1.07								
Force_50 (kg)	I	21.88 ± 6.6	2.35	1.88–3.15	6.50	10.49	8.43–14.09	0.939	0.877–0.969	Excellent
	II	22.84 ± 7.18								
Force_100 (kg)	I	28.79 ± 7.64	2.27	1.82–3.05	6.29	7.79	6.26–10.46	0.956	0.911–0.978	Excellent
	II	29.5 ± 7.92								
Force_250 (kg)	I	34.26 ± 7.62	1.24	1–1.67	3.45	3.60	2.89–4.84	0.987	0.974–0.994	Excellent
	II	34.81 ± 7.97								

SD = standard deviation; SEM = standard error of measurement; CI = confidence interval; ICC = intraclass correlation coefficient; CV = coefficient of variation; RFD = rate of force development; Imp = impulse.

## Data Availability

The data presented in this study are available on request from the corresponding author. The data are not publicly available due to privacy and ethical restrictions.

## Supplementary Materials

The following supporting information can be downloaded at: https://www.mdpi.com/article/10.3390/muscles5020024/s1, Table S1: Tests for systematic bias in intraday (trial effects) and interday (day-to-day) comparisons.

## References

[1] Filingeri D., Bianco A., Palma A. (2013). Handgrip strength: A predictive indicator of upper body maximal strength?. J. Sports Med. Phys. Fit..

[2] Maurya P.S., Sisneros K.P., Johnson E.B., Palmer T.B. (2023). Reliability of handgrip strength measurements and their relationship with muscle power. J. Sports Med. Phys. Fit..

[3] Schulz S.V.W., Wizani L., Matits L., Schwarz E., Wiedemann P., Bizjak D.A., Jerg A., Kirsten J., Henze A.-S. (2025). Handgrip strength in elite youth football: Potential for performance prediction and the moderating effects of age and maturation. Front. Sports Act Living.

[4] Sáez de Asteasu M.L., Cadore E.L., Steffens T., Blanco-Rambo E., Molinari T., Bandeira-Guimaraes M., Izquierdo M., Pietta-Dias C. (2025). Moderation Effect of Handgrip Strength on Cognition and Functional Independence Associations in Adults Over 90 Years. J. Cachexia Sarcopenia Muscle.

[5] Vaishya R., Misra A., Vaish A., Ursino N., D’Ambrosi R. (2024). Hand grip strength as a proposed new vital sign of health: A narrative review of evidences. J. Health Popul. Nutr..

[6] López-Bueno R., Andersen L.L., Koyanagi A., Núñez-Cortés R., Calatayud J., Casaña J., Cruz B.d.P. (2022). Thresholds of handgrip strength for all-cause, cancer, and cardiovascular mortality: A systematic review with dose-response meta-analysis. Ageing Res. Rev..

[7] Chai L., Zhang D., Fan J. (2024). Comparison of grip strength measurements for predicting all-cause mortality among adults aged 20+ years from the NHANES 2011–2014. Sci. Rep..

[8] Chang M., Geirsdottir O.G., Eymundsdottir H., Thorsdottir I., Jonsson P.V., Ramel A. (2022). Association between baseline handgrip strength and cognitive function assessed before and after a 12-week resistance exercise intervention among community-living older adults. Aging Health Res..

[9] Ruprai R., Tajpuriya S., Mishra N. (2016). Handgrip strength as determinant of upper body strength/physical fitness: A comparative study among individuals performing gymnastics (ring athletes) and gymnasium (powerlifters). Int. J. Med. Sci. Public Health.

[10] Szaflik P., Zadoń H., Michnik R., Nowakowska-Lipiec K. (2025). Handgrip Strength as an Indicator of Overall Strength and Functional Performance—Systematic Review. Appl. Sci..

[11] Sarvaiya P., Puntambekar A. (2022). Correlation between Lower Limb Power and Agility on Hand Grip Strength in three Different Positions in Young Fencers. Int. J. Innov. Res. Med. Sci..

[12] Park M.H., Park D., Kim K., Kang M.B. (2025). The relationship between grip strength, back strength and performance in vertical and standing long jump in athletes. Isokinet. Exerc. Sci..

[13] Aagaard P., Simonsen E.B., Andersen J.L., Magnusson P., Dyhre-Poulsen P. (2002). Increased rate of force development and neural drive of human skeletal muscle following resistance training. J. Appl. Physiol..

[14] Schumann M., Feuerbacher J.F., Alcazar J., Behm D.G., Granacher U., Häkkinen K., Walker S. (2024). Comment on: “Explosive is not a Term Defined in the International System of Units and Should not be Used to Describe Neuromuscular Performance.”. Int. J. Strength Cond..

[15] Maffiuletti N.A.N., Aagaard P., Blazevich A., Folland J.J., Tillin N., Duchateau J. (2016). Rate of force development: Physiological and methodological considerations. Eur. J. Appl. Physiol..

[16] McBride J.M., Bauer E.C., Kaufmann N.C., Triplett N.T., Shanely R.A. (2025). Handgrip Strength Associated with Leg Strength, Power, and Muscle Mass in 18–64-Year-Old Males and Females. J. Strength Cond. Res..

[17] Curovic I., Rhodes D., Alexander J., Harper D.J. (2024). Vertical Strength Transfer Phenomenon Between Upper Body and Lower Body Exercise: Systematic Scoping Review. Sports Med..

[18] Cronin J., Lawton T., Harris N., Kilding A., McMaster D.T. (2017). A Brief Review of Handgrip Strength and Sport Performance. J. Strength Cond. Res..

[19] Currell K., Jeukendrup A.E. (2008). Validity, Reliability and Sensitivity of Measures of Sporting Performance. Sports Med..

[20] Benton M.J., Spicher J.M., Silva-Smith A.L. (2022). Validity and reliability of handgrip dynamometry in older adults: A comparison of two widely used dynamometers. PLoS ONE.

[21] de Almeida M.B., Oliveira C., Ornelas G., Soares T., Souto J., Póvoa A.R., Ferreira L.M.A., Ricci-Vitor A.L. (2023). Intra-Rater and Inter-Rater Reliability of the Kinvent Hand-Held Dynamometer in Young Adults. Med. Sci. Forum.

[22] Khartabil A.L., Palmer T.B. (2025). Reliability of handgrip strength measurements and their relationship with physical performance outcomes in older women. Int. J. Exerc. Sci. Conf. Proc..

[23] Nikodelis T., Savvoulidis S., Athanasakis P., Chalitsios C., Loizidis T. (2021). Comparative Study of Validity and Reliability of Two Handgrip Dynamometers: K-Force Grip and Jamar. Biomechanics.

[24] van Iperen I.D., Stegink D., Tempert-de Haan B.L., Flim M., van der Stoep R., Spronk P.E. (2025). Reliability, validity and usability of the K-force^®^ grip dynamometer to evaluate handgrip-strength in patients with intensive care unit-acquired weakness. Clin. Rehabil..

[25] Mokkink L.B., de Vet H., Diemeer S., Eekhout I. (2023). Sample size recommendations for studies on reliability and measurement error: An online application based on simulation studies. Health Serv. Outcomes Res. Method.

[26] Maurya P., Sisneros K., Johnson E., Palmer T. (2023). Handgrip Peak Force and Rate of Force Development Measurements: Are They Reliable and Do They Correlate with Vertical Jump Power?. Int. J. Exerc. Sci. Conf. Proc..

[27] Koo T.K., Li M.Y. (2016). A Guideline of Selecting and Reporting Intraclass Correlation Coefficients for Reliability Research. J. Chiropr. Med..

[28] Weir J.P. (2005). Quantifying test-retest reliability using the intraclass correlation coefficient and the SEM. J. Strength Cond. Res..

[29] Hopkins W.G. (2000). Measures of reliability in sports medicine and science. Sports Med..

[30] Jenkins N.D., Buckner S.L., Bergstrom H.C., Cochrane K.C., Goldsmith J.A., Housh T.J., Johnson G.O., Schmidt R.J., Cramer J.T. (2014). Reliability and relationships among handgrip strength, leg extensor strength and power, and balance in older men. Exp. Gerontol..

[31] Gandevia S.C. (2001). Spinal and supraspinal factors in human muscle fatigue. Physiol. Rev..

[32] Ewing J.L., Stull G.A. (1984). Rate of Force Development in the Handgripping Muscles by Females as a Function of Fatigue Level. Res. Q. Exerc. Sport.

[33] Labott B.K., Donath L. (2023). Agility performance in healthy older adults is associated with handgrip strength and force development: Results from a 1-year randomized controlled trial. Eur. Geriatr. Med..

[34] Sisneros K., Maurya P., Johnson E., Ford B., Palmer T.Y. (2023). Age-related differences in vertical jump height and handgrip strength measurements. Acta Bioeng. Biomech..

[35] Langford M., Hackney K.J., Andrew S., Batesole J., Berntson M., Black K., Hoang T., Klawitter L., Kraemer W.J., McGrath R. (2024). The Relationships Between Upper and Lower Extremity Muscle Strength, Rate of Force Development, and Fatigue in Adults. Int. J. Exerc. Sci..

[36] Bohannon R.W. (2019). Minimal clinically important difference for grip strength: A systematic review. J. Phys. Ther. Sci..

