# Supplementary material for: Intraday and Interday Reliability of Maximal and Explosive Handgrip Force–Time Metrics Using the Kinvent K-Grip Handheld Dynamometer
Source: Muscles. 2026 Mar 25;5(2):24. doi: 10.3390/muscles5020024 (PMC13108196; doi:10.3390/muscles5020024)
Supplement: Supplementary file 1 [file muscles-05-00024-s001.zip › muscles-4151008-supplementary.pdf]

Supplementary Table S1. Tests for systematic bias in intraday (trial effects) and interday (day-to-day) comparisons.

Variable	Intraday ANOVA p (Right)	Intraday ANOVA p (Left)	Interday paired t-test p
Peak force	0.303	>0.05	>0.05
Mean force	0.231	>0.05	>0.05
Peak RFD	0.393	>0.05	>0.05
$RFD_{0–50}$	0.489	>0.05	>0.05
$RFD_{0–100}$	0.163	>0.05	>0.05
$RFD_{0–150}$	0.914	>0.05	>0.05
$RFD_{0–200}$	0.819	>0.05	>0.05
$Impulse_{0–50}$	0.310	>0.05	>0.05
$Impulse_{0–150}$	0.287	>0.05	>0.05
$Impulse_{0–200}$	0.358	>0.05	>0.05
$Force_{50}$	0.437	>0.05	>0.05
$Force_{100}$	0.634	>0.05	>0.05
$Force_{250}$	0.306	>0.05	>0.05
